# Hyperpolarization-Activated Currents and Subthreshold Resonance in Granule Cells of the Olfactory Bulb

**DOI:** 10.1523/ENEURO.0197-16.2016

**Published:** 2016-11-04

**Authors:** Ruilong Hu, Katie A. Ferguson, Christina B. Whiteus, Dimphna H. Meijer, Ricardo C. Araneda

**Affiliations:** 1 Department of Biology, University of Maryland, College Park, Maryland 20742; 2 Neurobiology Course, Marine Biology Laboratory, Woods Hole, Massachusetts 02543

**Keywords:** granule cell, HCN, neurogenesis, olfactory bulb, resonance

## Abstract

An important contribution to neural circuit oscillatory dynamics is the ongoing activation and inactivation of hyperpolarization-activated currents (*I*_h_). Network synchrony dynamics play an important role in the initial processing of odor signals by the main olfactory bulb (MOB) and accessory olfactory bulb (AOB). In the mouse olfactory bulb, we show that *I*_h_ is present in granule cells (GCs), the most prominent inhibitory neuron in the olfactory bulb, and that *I*_h_ underlies subthreshold resonance in GCs. In accord with the properties of *I*_h_, the currents exhibited sensitivity to changes in extracellular K^+^ concentration and ZD7288 (4-ethylphenylamino-1,2-dimethyl-6-methylaminopyrimidin chloride), a blocker of *I*_h_. ZD7288 also caused GCs to hyperpolarize and increase their input resistance, suggesting that *I*_h_ is active at rest in GCs. The inclusion of cAMP in the intracellular solution shifted the activation of *I*_h_ to less negative potentials in the MOB, but not in the AOB, suggesting that channels with different subunit composition mediate *I*_h_ in these regions. Furthermore, we show that mature GCs exhibit *I*_h_-dependent subthreshold resonance in the theta frequency range (4–12 Hz). Another inhibitory subtype in the MOB, the periglomerular cells, exhibited *I*_h_-dependent subthreshold resonance in the delta range (1–4 Hz), while principal neurons, the mitral cells, do not exhibit *I*_h_-dependent subthreshold resonance. Importantly, *I*_h_ size, as well as the strength and frequency of resonance in GCs, exhibited a postnatal developmental progression, suggesting that this development of *I*_h_ in GCs may differentially contribute to their integration of sensory input and contribution to oscillatory circuit dynamics.

## Significance Statement

The hyperpolarization-activated current (*I*_h_) plays an essential role in neuronal function and network oscillatory dynamics throughout the nervous system. Network synchrony dynamics are an important component of the initial processing of odor signals in the olfactory bulb (OB). Here we provide new evidence that granule cells (GCs), the major inhibitory subtype in the OB, exhibit an *I*_h_-dependent subthreshold resonance in the theta frequency range (4–12 Hz). Notably, *I*_h_ size and the strength of subthreshold resonance in GCs exhibited a postnatal developmental progression, suggesting that this development of *I*_h_ in GCs may differentially contribute to their integration of sensory input and contribution to oscillatory circuit dynamics.

## Introduction

Hyperpolarization-activated cyclic nucleotide-gated (HCN) channels play an essential role in neuronal function and network oscillatory dynamics, including the control of membrane excitability and integration of synaptic inputs, the generation of membrane potential oscillations, and subthreshold resonance of neurons (McCormick and Pape, 1990; Magee, 1998; Fan et al., 2005; George et al., 2009; Hu et al., 2009). Moreover, the voltage sensitivity and kinetic properties of the hyperpolarization-activated current (*I*_h_), is dependent on the subunit composition of the channels (HCN1–HCN4), which confers differing sensitivities to cyclic nucleotides, providing a rich heterogeneity to the contribution of these channels to network function (Kaupp and Seifert, 2001; Wainger et al., 2001). Thus, changes in intracellular cAMP concentration in response to neuronal metabolic state or to the action of neuromodulators can affect the *I*_h_ and, in turn, alter membrane potential oscillations of pacemaker cells in the brain and in the heart (Pape and McCormick, 1989; Valsecchi et al., 2013).

In olfaction, initial processing of odor signals occurs through two parallel, albeit complementary, pathways in the olfactory bulb (OB). In general, the main OB (MOB) receives input from the nasal epithelium and is largely tuned to environmental odors, whereas the accessory OB (AOB) receives input from the vomeronasal organ (VNO) and is largely responsible for the detection of semiochemicals (Shepherd, 1972; Keverne, 1999; Lledo et al., 2005). In the MOB, network synchrony is a substrate to important aspects of olfactory coding, such as sparsening and feature binding of odor representations (Brody and Hopfield, 2003; Binns and Brennan, 2005; Osinski and Kay, 2016), and network synchrony may also play a role in pheromonal processing by the AOB (Binns and Brennan, 2005; Leszkowicz et al., 2012). This network synchrony arises from the activity of dendrodendritic synapses between a large number of intrinsic inhibitory neurons, including the granule cells (GCs) and periglomerular cells (PGCs), and the output neurons, the mitral cells (MCs) and tufted cells (Lagier et al., 2004; Lepousez and Lledo, 2013; Kay, 2014; Gschwend et al., 2015). In the MOB, network oscillations in the theta frequency (4–12 Hz), entrained by the respiratory cycle, are in part mediated by PGCs (Fukunaga et al., 2014), while gamma oscillations (40–80 Hz) are mediated by GCs (Lagier et al., 2004; Fukunaga et al., 2014; Kay, 2014).

Surprisingly, despite the influence of inhibitory neurons in promoting network oscillations in the OB, the presence and contribution of *I*_h_ to GC excitability has not been addressed. Here, using whole-cell patch-clamp recordings, we show that most GCs of the OB exhibit *I*_h_. Targeted recording of postnatally labeled neurons indicated that the contribution of *I*_h_ to GC physiology increases throughout postnatal development, leveling between 4 and 6 weeks after birth. In the presence of ZD7288 (4-ethylphenylamino-1,2-dimethyl-6-methylaminopyrimidin chloride), an *I*_h_ blocker, GCs hyperpolarized and their input resistance increased, suggesting that, at rest, *I*_h_ contributes to the excitability and passive properties of GCs. Importantly, mature MOB GCs and PGCs exhibited an *I*_h_-dependent subthreshold resonance with resonant frequencies in the delta and theta ranges (1–12 Hz). These results suggest that the expression of *I*_h_ and subthreshold resonance in inhibitory neurons may impart unique features to odor processing in the OB, and facilitate oscillatory network activity in both the main olfactory and vomeronasal systems.

## Materials and Methods

### Animals

All experiments were conducted following the guidelines of the institutional animal care and use committee of the University of Maryland and the Marine Biology Laboratory. Experiments were performed on wild-type (C57BL/6) mice of either sex, ranging in age from postnatal day 15 (P15) to 60 (P60).

### Electroporation

To label postnatally born neurons with the green fluorescent protein (GFP), mice (P1–P4) were anesthetized by hypothermia and 2 μl (4 μg/μl) of pCAG-GFP plasmid (Addgene) was injected into the lateral ventricle. Immediately after the injection, twizzer-type electrodes (BTX) were placed on the sides of the head for electroporation (5 × 100 V pulses of 50 ms) using an ECM 830 Electro Square Electroporation System (BTX). Recordings of GCs were conducted at least 1 week postelectroporation, and onward, in cells identified by the expression of GFP under a fluorescent microscope.

### Slice preparation

Experiments were performed in OB slices using methods previously described (Smith et al, 2015). Briefly, brain slices were prepared in an oxygenated ice-cold artificial CSF (ACSF) containing low Ca^2+^ (0.5 mm) and high Mg^2+^ (6 mm). Sagittal sections of the OB (250 µm) were then transferred to an incubation chamber containing normal ACSF (see below) and were left to recuperate for at least 30 min at 35°C until the recordings. In all experiments, unless otherwise stated, the extracellular solution is ACSF of the following composition (in mm): 125 NaCl, 26 NaHCO3, 1.25 NaH2PO4, 2.5 KCl, 2 CaCl2, 1 MgCl2, 1 myoinositol, 0.4 ascorbic acid, 2 Na-pyruvate, and 15 glucose, continuously oxygenated (95% O_2_-5% CO_2_), which produced a pH of 7.4 and an osmolarity of 305 mOsm.

### Electrophysiological recordings

Neurons were visualized using an Olympus BX51W1 microscope and were recorded using a dual EPC10 amplifier (HEKA) in voltage-clamp and current-clamp modes. In a subset of the experiments, to visualize and confirm the identity and morphology of GCs, the fluorophore Alexa Fluor 594 (red) was included in the recording pipette solution (20 μm). Electrical stimulations and recordings were performed using the PatchMaster software. Experiments were performed at room temperature, or at 32 ± 2°C, using the TC-342B Automatic Temperature Controller (Warner Instruments) with a perfusion speed of 2–3 ml/min. Cells were patched using standard patch pipettes (resistance, 4–8 MΩ). The membrane potential was not corrected for junction potential. Although our experiments in whole-cell recordings precluded an accurate measurement of membrane potential (*V*_m_), right after rupturing the seal, the *V*_m_ in GCs of the MOB was −71.9 ± 1.5 mV (*n* = 26) and −64.4 ± 1.5 mV (*n* = 5) in the AOB. These measurements are in agreement with those from previous studies indicating that GCs maintain a hyperpolarized *V*_m_ in the slice preparation (Cang and Isaacson, 2003); Egger et al., 2005). Therefore, unless otherwise indicated, voltage-clamp and current-clamp experiments in GCs were conducted at −60 or −70 mV.

### Solutions and pharmacological agents

In whole-cell recordings, the internal solution had the following composition (in mm): 120 K-gluconate, 10 Na-Gluconate, 4 NaCl, 10 HEPES-K, 10 Na phosphocreatine, 2 Na-ATP, 4 Mg-ATP, and 0.3 GTP adjusted to pH 7.3 with KOH. This internal solution, which contains Na, was used in order to compare the physiology of GCs with previous work published from our laboratory (Smith et al., 2015). In some experiments, 0.5 mm cAMP was added to the internal solution. The osmolarity of the internal solutions was adjusted to 290–305 mOsm. For experiments using high extracellular K^+^, the ACSF contained 25 mm KCl and 102.5 mm NaCl. Drugs were prepared fresh from stocks and diluted into the external solution; ZD7288 was purchased from Tocris Cookson, and it was applied at 30 μm.

### Data analysis

All electrophysiological recordings were analyzed in MATLAB (MathWorks). In order to isolate *I*_h_, leak currents were subtracted from the voltage–current relationships using multiples of a small voltage step (−60 to −65 mV) elicited at the end of each voltage-clamp trace. Recorded GCs exhibited heterogeneity in physiological properties, such as input resistance and capacitance, presumably due to the presence of a heterogeneous population that included mature and immature cells. To compensate for these differences, in some instances (i.e., high K^+^ or ZD288) *I*_h_ was normalized to the current elicited by a voltage step from −60 to −130 mV.

Voltage dependency was calculated by fitting the tail-current maximum values to a Boltzmann function of the following form (Ludwig et al., 1998; Dibattista et al., 2008):

$$I=I_{\max}/\{1+\exp[(V-V_{half})/k_{s}]\},$$

where *I*_max_ is the maximal current, *V* is the prepulse potential, *V*_half_ is the half-activation potential of the current, and *k*_s_ is the slope factor. The current (*I*) values were measured as the peak current obtained with a step to −130 mV from the prepulse potential (see Fig. 2*A*, inset) and were then normalized to the fitted *I*_max_ value for each cell. The activation time constant (τ) for *I*_h_ was determined by fitting a single exponential function to the raw voltage trace when stepping from −60 to −130 mV.

The sag potential was calculated from the voltage deflection elicited by a negative current pulse, and corresponds to the difference between the peak minimum voltage and the *V*_m_ upon reaching a steady state (after 1 s). The change in membrane potential (Δ*V*_m_) in response to ZD7288 was measured as the difference between the baseline *V*_m_ value (before the administration of the drug) and the *V*_m_ value at the peak of the response, which, under our perfusion speed, occurred within ∼6–10 min. Subthreshold resonance was measured using a standard impedance amplitude protocol (ZAP), in which a stimulus of sinusoidal current of constant amplitude and exponentially increasing frequency (0.2–20 Hz) is injected into the cell. The ZAP protocol was obtained at different negative potentials (−60 to −90 mV), which are correspondingly indicated throughout the text. The exponential ZAP protocol we used works best on cells with lower resonant frequencies; therefore, we also confirmed our findings in mature cells using a linear ZAP protocol (Hu et al., 2002; Narayanan and Johnston, 2007), allowing us to better sample higher frequencies (data not shown). The impedance profile was calculated as the magnitude of the ratio of the fast Fourier transforms of the voltage response and current input (Gutfreund et al., 1995; Hutcheon and Yarom, 2000; Vera et al., 2014) as follows:

Z(f)=|FFT[V(t)]/FFT[/(t)]|.

The impedance profile is smoothed, and the resonant frequency (*f*_res_) is the frequency at which the maximal impedance value occurs. If the peak value is not clear, the impedance profile is fit with a quadratic function to provide an estimate. The strength of resonance (*Q* factor) is calculated as the ratio between the maximal impedance (|*Z*(*f*_res_)|) and the lowest frequency impedance values (|*Z*(*f*_low_)|; 0.5 Hz). The cell is considered resonant if *f*_res_ > *f*_low_ and *Q* > 1. Statistical significance was determined by a Student’s *t* test, Mann–Whitney *U* test, one-way ANOVA, logistic regression, or linear regression.

## Results

### *I*_h_ is present in GCs of the AOB and MOB

**Figure 1.**
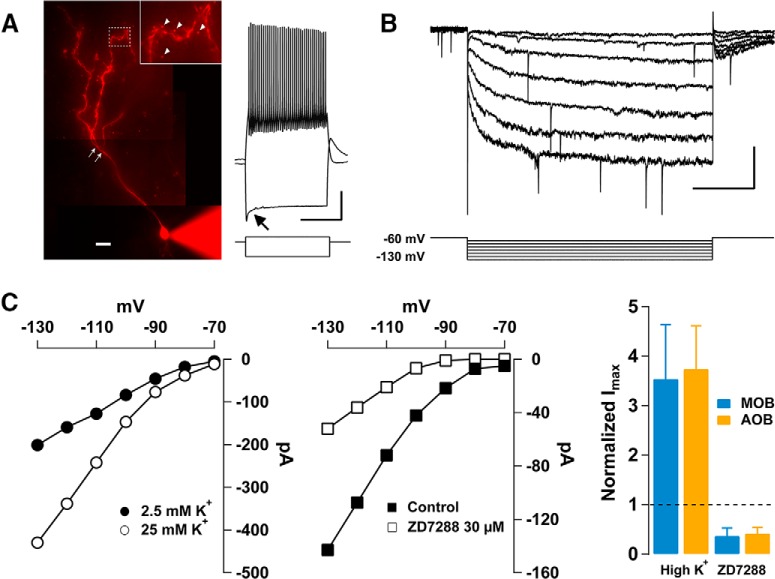
Properties of hyperpolarization-activated cation currents in GCs of the OB. ***A***, Left, Image of a GC recorded in the AOB, with Alexa Fluor 594 in the pipette. Mature GCs are characterized by the presence of branches in the apical dendrite (white arrows), and prominent dendritic spines (inset, arrow heads). Scale bar, 20 μm. Right, Current-clamp response of the GC shown on the left. A hyperpolarizing current step (−60 pA) produces a sag in the *V*_m_ (arrow), while a depolarizing current step (20 pA) elicits a train of action potentials. The resting *V*_m_ in this cell is −60 mV. Calibration: 20 mV and 1 s. ***B***, Hyperpolarizing voltage steps from −60 to −130 mV (10 mV steps) elicited a slowly developing hyperpolarization-activated inward current. Calibration: 500 ms and 50 pA. ***C***, Left, Current–voltage (*I–V*) relationship for *I*_h_ in an AOB GC. Raising the external K^+^ concentration from 2.5 mm (filled circles) to 25 mm (empty circles) increased the inward current at each potential. Middle, *I–V* relationship of an AOB GC in control (filled squares) and in the presence of the *I*_h_ blocker ZD7288 (30 μm, empty squares); at all potentials ZD7288 reduced *I*_h_. Right, Summary plot showing the effect of high K^+^ and ZD7288 on the normalized mean *I*_h_ at −130 mV. In the MOB (blue) and AOB (orange), high K^+^ produced at least a threefold increase in the current. Similarly, ZD7288 produced a >60% decrease in *I*_h_ in both regions.

We conducted whole-cell patch-clamp recordings in GCs of the MOB and AOB. In both regions, GCs are located in easily identified layers and exhibit characteristic morphology, including the presence of basal dendrites and a single apical dendrite, which bifurcates into several branches, populated by prominent dendritic spines (Fig. 1*A*; Price and Powell, 1970; Larriva-Sahd, 2008). In voltage clamp (Fig. 1*B*), hyperpolarizing steps from −60 to −130 mV revealed a slowly developing inward current, with characteristics of *I*_h_, that occurred in the majority of GCs [∼96% in the AOB (80 of 83 GCs) and ∼90% in the MOB (106 of 118 GCs)]. In agreement with the properties of *I*_h_, the peak amplitude of the inward current was enhanced by approximately threefold by increasing the external K^+^ concentration from 2.5 to 25 mm (Fig. 1*C*). Thus, the normalized mean currents in high K^+^ were 3.7 ± 0.9 (*n* = 5; Mann–Whitney *U* test, *p* < 0.01) in the AOB and 3.5 ± 1.1 in the MOB (*n* = 4; Mann–Whitney *U* test, *p* < 0.01). Importantly, in the presence of the selective *I*_h_ blocker ZD7288 (30 μm; see Materials and Methods), the inward current was reduced by over 60% (Fig. 1*C*). The normalized mean current in the presence of ZD7288 was 0.4 ± 0.1 in the AOB (*n* = 4; Mann–Whitney *U* test, *p* < 0.03) and 0.36 ± 0.16 in the MOB (*n* = 7; Mann–Whitney *U* test, *p* < 0.001).

**Figure 2.**
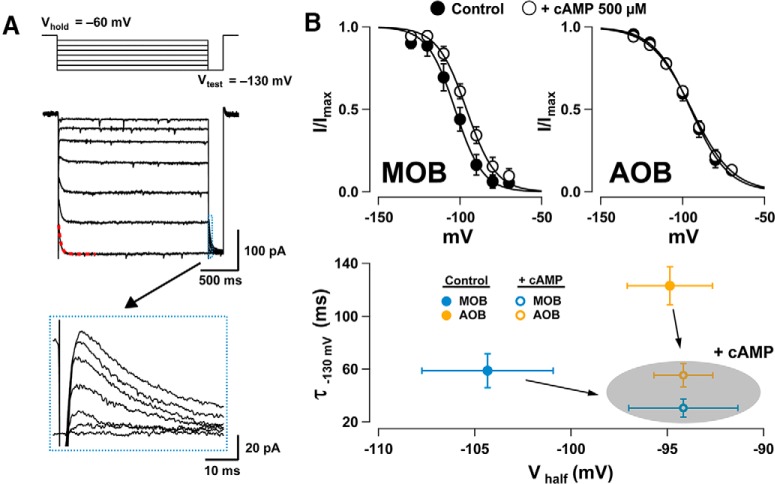
Voltage dependency of *I*_h_ and sensitivity to intracellular cAMP. ***A***, Voltage-clamp responses in an MOB GC with added cAMP in the internal solution showing *I*_h_ elicited by 10 mV hyperpolarizing steps from −60 to −130 mV. At the end of each step, a test pulse to −130 mV (*V*_test_) was elicited to measure the maximal current at each potential (traces shown in the bottom box inset). The current amplitudes measured at the peak were normalized to *I*_max_ and fitted to a Boltzmann function (see Materials and Methods). The τ value was estimated by fitting the current elicited by stepping from −60 to −130 mV (red dotted line, 25.5 ms for this trace). ***B***, Top, Boltzmann fits for *I*_h_ in GCs of the MOB (left) and AOB (right) in recordings conducted with internal solutions without cAMP (filled circles) or containing cAMP (0.5 mm, empty circles). Bottom, Summary plot comparing the cAMP sensitivity of *V*_half_ and τ in AOB GCs (orange) and MOB GCs (blue). In the presence of cAMP, the *V*_half_ was shifted to more positive potentials in MOB CGs, while in the AOB, τ was reduced (shaded oval).

Different subunit composition in the tetrameric HCN channels confers distinct cyclic nucleotide sensitivity to *I*_h_, which in turn influences the voltage sensitivity and kinetics of the channels (Craven and Zagotta, 2006; Benarroch, 2013). Therefore, we compared the properties of *I*_h_ by recording GCs with and without cAMP (0.5 mm) in the internal solution, determining the kinetics of *I*_h_ activation and maximal current elicited in voltage clamp (Fig. 2*A*). Analysis of the voltage dependency using a Boltzmann fit (see Materials and Methods) and the kinetics of activation of *I*_h_ revealed significant differences between GCs in the AOB and MOB. In the absence of cAMP, the activation of *I*_h_ occurred at more positive potentials in the AOB than in the MOB; the *V*_half_ of the activation of *I*_h_ in AOB was −94.8 ± 4 mV (*n* = 15), while in the MOB *V*_half_ was −103 ± 6 mV (*n* = 10; AOB vs MOB, Student’s *t* test, *p* < 0.03). Furthermore, in the absence of cAMP, the activation of *I*_h_ was slower in the AOB than in the MOB (Fig. 2*B*; τ = 123 ± 14 ms vs τ = 58.8 ± 12.9 ms, respectively; Student’s *t* test, *p* < 0.007). Interestingly, adding cAMP to the internal solution shifted *V*_half_ by ∼10 mV in the MOB (−94 ± 5 mV, *n* = 11, Student’s *t* test, *p* < 0.04), while it produced no significant effect in the AOB (−94.5 ± 3 mV, *n* = 12; Student’s *t* test, *p* > 0.4). In contrast, τ decreased by approximately half in both regions (AOB: 55.3 ± 8.7 ms; Student’s *t* test, *p* < 0.001; MOB: 30 ± 6.8 ms; Student’s *t* test, *p* < 0.03). Together, these findings indicate that the sensitivity to cAMP is region specific and suggest that GCs may express HCN channels with different subunit composition in these regions.

**Figure 3.**
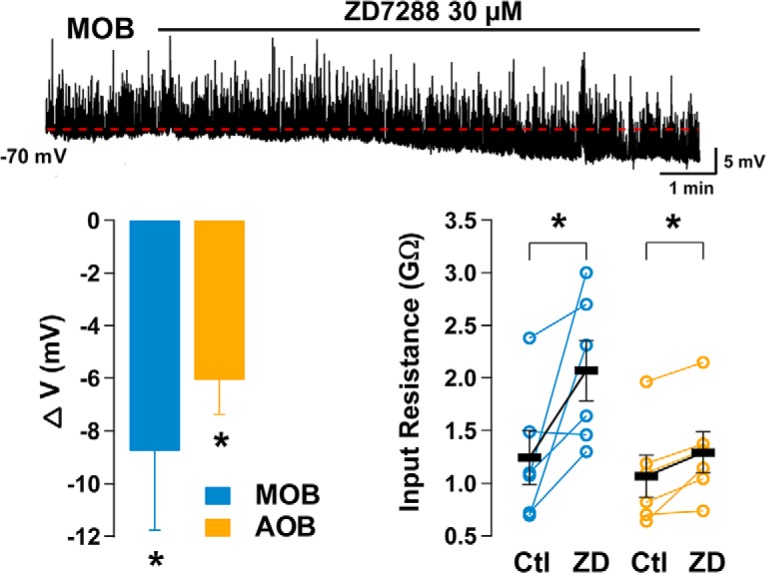
I_h_ contributes to the intrinsic properties of GCs. Top trace, Current-clamp responses in an MOB GC. Bath perfusion of ZD7288 produces a hyperpolarization (red dotted line). Spontaneous postsynaptic potentials appear as upward spikes. Bottom left, Summary of the effects of ZD7288 on *V*_m_. Application of ZD7288 reduces the membrane potential of GCs of the MOB and AOB. Right, Pharmacological block of *I*_h_ significantly increases the input resistance of GCs measured from a holding potential of −60 mV with a negative current step.

The voltage dependency of *I*_h_ in GCs suggests that it could be active at resting *V*_m_ (see Materials and Methods). In agreement with this possibility, in current-clamp experiments bath perfusion of ZD7288 invariably produced a hyperpolarization in GCs (Fig. 3). Blocking *I*_h_ reduced the membrane potential by 6 ± 1.3 mV in the AOB (orange, *n* = 6; Student’s *t* test, *p* < 0.005) and by 8.8 ± 3.7 mV in the MOB (blue, *n* = 5; Student’s *t* test, *p* < 0.04). This effect of ZD7288 was still present in the presence of blockers of fast synaptic transmission (APV, 100 μm; CNQX, 10 μm; and gabazine, 10 μm), suggesting a direct effect on GCs. Application of ZD7288 reduced the membrane potential by 3.7 ± 1 mV in AOB GCs (*n* = 4; Student’s *t* test, *p* < 0.007) and 6.9 ± 3.3 mV in MOB GCs (*n* = 4; Student’s *t* test, *p* < 0.05). In addition, ZD7288 produced a significant increase in GCs input resistance (*R*_i_) in both regions (Fig. 3; AOB; 1.04 ± 0.20 vs 1.27 ± 0.19 GΩ; Student’s *t* test, *p* < 0.01; MOB 1.24 ± 0.25 vs 2.06 ± 0.28 GΩ; Student’s *t* test, *p* < 0.05).

### GCs exhibit subthreshold resonance that depends on *I*_h_

**Figure 4.**
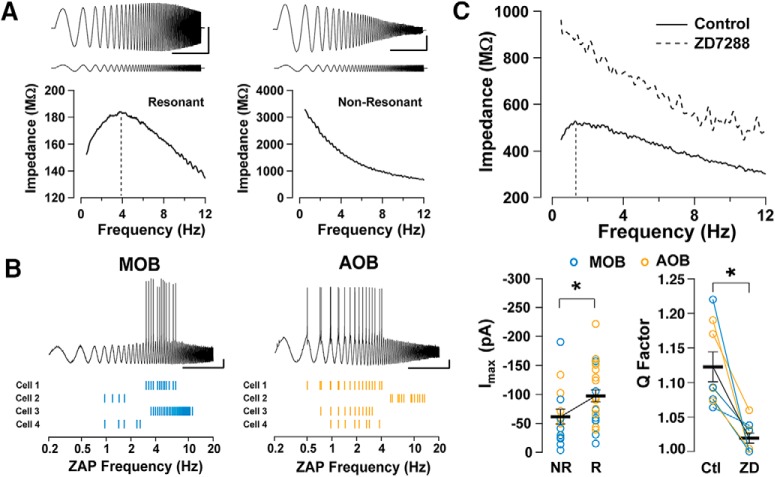
Contribution of *I*_h_ to subthreshold resonance in GCs. ***A***, Top, Voltage responses (top traces) to a ZAP current stimulus (bottom traces) in MOB GCs in a resonant (left) and, a nonresonant (right) cell. Bottom, Impedance profiles for the cells shown above. In the resonant cell (left), the *Q* value is 1.20 and the *f*_res_ value is 3.88 Hz; whereas, for the nonresonant cell (right), *f*_res_ = *f*_low_ and the *Q* = 1. Calibration: 5 s and 10 mV [with a 50 pA (left) and 5 pA (right) ZAP stimulus amplitude]. The *V*_m_ for both cells is −80 mV. ***B***, ZAP stimulus applied to resonant GCs in the MOB (top) and AOB (bottom); the current amplitude was adjusted to elicit action potentials, which occurred around the resonant frequency of the cell. ***C***, Top, The impedance profile of an AOB GC that exhibits subthreshold resonance in control conditions and, in the presence of ZD7288. In control conditions, *Q* = 1.21 and *f*_res_ = 1.3 Hz (black line). In the presence of ZD7288, the resistance of the GC increases, the resonance is abolished, and the impedance profile resembles that of a nonresonant cell (*Q* = 1). Bottom left, Summary plot showing the distribution of values for maximal *I*_h_ (measured at −130 mV) for MOB (blue, *n* = 19) and AOB (orange, *n* = 21) GCs; resonant cells have larger *I*_h_ values. Bottom right, The *Q* factor significantly decreases after perfusion of ZD7288 in the AOB (*n* = 4) and MOB (*n* = 4).

In other brain regions, *I*_h_ plays a critical role in generating subthreshold resonance of neurons, which in turn contributes to network oscillatory properties (Hu et al., 2002; Narayanan and Johnston, 2007; van Brederode and Berger, 2011; Vera et al., 2014). Therefore, we examined subthreshold resonance in GCs that exhibited *I*_h_, using a sinusoidal current stimulus of increasing frequency (ZAP stimulus; see Materials and Methods) in animals older than P30. Surprisingly, we observed a significant heterogeneity in responses to the ZAP stimulus among GCs, with a mixed population of resonant and nonresonant cells (Fig. 4*A*). The percentage of resonant cells in the AOB was ∼71% (15 of 21 cells), and in the MOB was ∼52% (10 of 19 cells). In addition, among all resonant cells (AOB plus MOB), the response to the ZAP stimulus revealed heterogeneity in the *f*_res_, with cells distributed along the delta and theta range (data not shown). Importantly, this subthreshold resonance could affect the global excitability of GCs. Accordingly, when we adjusted the ZAP protocol to elicit action potentials in a subset of resonant MOB and AOB GCs, these action potentials occurred at a frequency within the range of *f*_res_ for each cell, with 79% of the action potentials falling within ±3 Hz relative to the resonant frequency of the cell (Fig. 4*B*).

The heterogeneity of subthreshold resonance that we observed prompted us to examine whether the size of *I*_h_ in GCs could contribute to these differences. Across the total population of GCs studied (AOB and MOB), resonant cells had larger *I*_h_ values than nonresonant cells, suggesting that *I*_h_ amplitude is a predictor of resonance (Fig. 4*C*; resonant cells, *I*_max_ = −97.5 ± 10.3 pA, *n* = 25; nonresonant cells, I_max_ = −61 ± 13.0 pA, *n* = 15; logistic regression, *p* < 0.02). Similarly, *I*_h_ amplitude is predictive of resonance strength (*I*_max_ vs *Q*, in a linear regression model, *p* < 0.02). In agreement with a larger *I*_h_ value, *R*_i_ was significantly lower in resonant cells than in nonresonant cells (1.14 ± 0.01 vs 1.61 ± 0.17 GΩ; logistic regression, *p* < 0.01). Notably, the subthreshold resonance was abolished in the presence of ZD7288, (Fig. 4*C*); the strength of resonance or *Q* factor (see Materials and Methods) was reduced from 1.12 ± 0.02 to 1.02 ± 0.01 in the presence of ZD7288 (*n* = 8; Student’s *t* test, *p* < 0.0005). Together, these results suggest that *I*_h_ is a major contributor to the expression of subthreshold resonance in AOB and MOB GCs.

**Figure 5.**
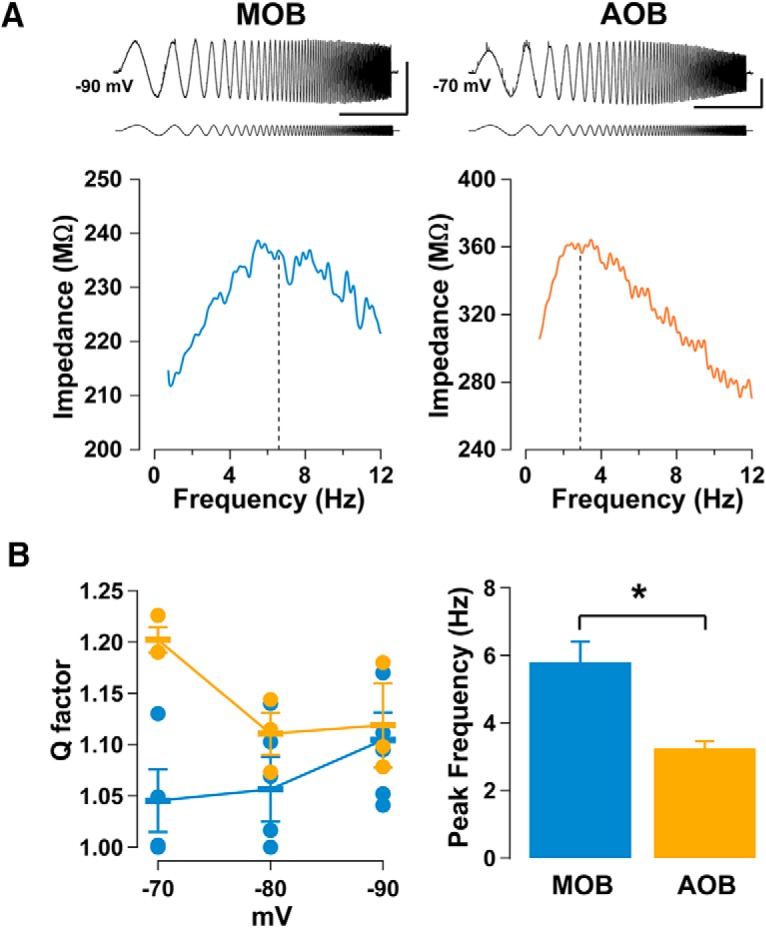
The subthreshold resonance in AOB and MOB GCs exhibits different voltage dependencies. ***A***, Top, ZAP protocol applied to 4-week-old mature GCs labeled with GFP, in the MOB (left) and AOB (right), along with their respective impedance profiles (bottom). For the MOB GC, *Q* is 1.17 and *f*_res_ is 6.77 Hz, indicated by the vertical black dashed line; for the AOB GC, *Q* is 1.19 and *f*_res_ is 2.89 Hz, indicated by the dashed line. Calibration: 10 mV and 5 s. The ZAP stimulus amplitude is 20 pA for the MOB and 30 pA for the AOB. Bottom, Summary plots for the voltage dependency of *Q*, and the peak frequencies in MOB (blue) and AOB (orange) GCs (*n* = 5 and 3, respectively). ***B***, Left, In MOB GCs, *Q* is strongest at more negative potentials, while in AOB GCs *Q* is larger at more positive potentials. Right, At −90 mV the average peak frequency in MOB GCs is significantly higher than in the AOB GCs (see Results).

To further characterize the subthreshold resonance in AOB and MOB GCs, we performed targeted recordings of postnatally labeled neurons (see Materials and Methods). After 4 weeks, GCs are fully integrated, and exhibit characteristic physiological and morphological properties (Petreanu and Alvarez-Buylla, 2002; Winner et al., 2002; Carleton et al., 2003; Lledo et al., 2006). At this age (Fig. 5*A*), the analysis of voltage dependency indicated that *f*_res_ in the AOB did not vary with voltage (−70 mV, 3.47 ± 0.37 Hz; −90 mV, 3.24 ± 0.21 Hz; *n* = 3; one-way ANOVA, *p* > 0.6). However, *f*_res_ was larger at more negative potentials in the MOB (−70 mV, *f*_res_ = 1.09 ± 0.45 Hz; −90 mV, *f*_res_ = 5.80 ± 0.60 Hz; *n* = 5; one-way ANOVA, *p* < 0.001). Thus, at −90 mV, the average peak frequency in MOB GCs is significantly higher than that in AOB GCs (Fig. 5*B*; Student’s *t* test, *p* < 0.03). Furthermore, although the values did not reach significance, there was a tendency for the strength of resonance to increase at a more negative potential in MOB GCs (Fig. 5*B*; −70 mV, *Q* = 1.04 ± 0.03; −90 mV, *Q* = 1.10 ± 0.02; *n* = 5; one-way ANOVA, *p* < 0.1), while we observed the opposite in the AOB (−70 mV, *Q* = 1.20 ± 0.01; −90 mV, *Q* = 1.12± 0.04; *n* = 4; one-way ANOVA, *p* < 0.03).

### Developmentally timed expression of *I*_h_ and subthreshold resonance in GCs in the MOB

**Figure 6.**
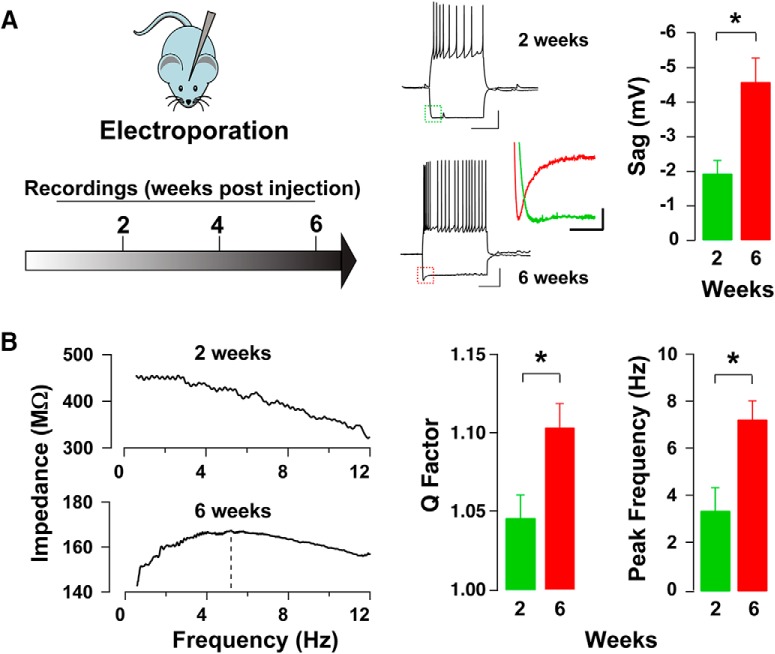
Postnatal development and subthreshold resonance in MOB GCs. ***A***, Left, Diagram of the time course used for labeling and recording from GCs at different stages of development. Mice were electroporated at P1, and recordings were conducted at different postnatal weeks. Right, Sample current-clamp traces from labeled GCs at 2 and 6 weeks; recorded cells exhibit robust action potentials. At both ages, a hyperpolarizing current step elicits an *I*_h_-mediated sag upon hyperpolarization (see inset). Calibration: 500 ms and 20 mV. The colored inset compares the sag amplitudes of the cells shown at 2 weeks (green) and 6 weeks (red). Calibration: 100 ms and 2 mV. The bar graph summarizes the change in sag amplitude between 2 and 6 weeks postlabeling. ***B***, Left, Example plots of the impedance profile at 2 and 6 weeks after electroporation obtained using a ZAP protocol, corresponding to the cells seen above in ***A***. At 2 weeks, *Q* is 1.02, while at 6 weeks *Q* is 1.14 and *f*_res_ is 5.18 Hz (dotted line). Right, Bar graphs showing the increase in *Q* factors and peak frequencies between 2 and 6 weeks.

GCs undergo a critical period and exhibit varied physiological properties over the course of development (Yamaguchi and Mori, 2005; Lledo et al., 2006; Lepousez et al., 2013); therefore, we compared *I*_h_ and subthreshold resonance expression in GCs at 2 and 6 weeks after birth in the MOB. As shown in Fig. 6*A*, we found that *I*_h_-mediated sag amplitude in GCs significantly increased between 2 weeks (*n* = 5) and 6 weeks (*n* = 5) after birth, (−1.9 ± 0.4 vs −4.6 ± 0.7 mV; Student’s *t* test, *p* < 0.006). Similarly, the *I*_h_ amplitude nearly doubled between 2 and 6 weeks, although it was not significant within our sampled population of cells (−121.1 ± 31.1 vs −215.9 ± 37.0 pA; Student’s *t* test, *p* < 0.09). Nevertheless, and in agreement with the developmental increase in *I*_h_-mediated sag amplitude, resonance in GCs increased as a function of cell age (Fig. 6*B*). Between 2 and 6 weeks, resonance strength increased from 1.05 ± 0.01 to 1.10 ± 0.01 (Student’s *t* test, *p* < 0.02). Interestingly, we also observed a developmental increase in *f*_res_. At 2 weeks, *f*_res_ was 3.44 ± 0.98 H, and at 6 weeks, 7.32 ± 0.79 Hz (Student’s *t* test, *p* < 0.02). Altogether, these results indicate that as GCs mature, their *f*_res_ becomes tuned towards the physiological breathing rates of mice (1–12 Hz).

### Subthreshold resonance in other OB subtypes

**Figure 7.**
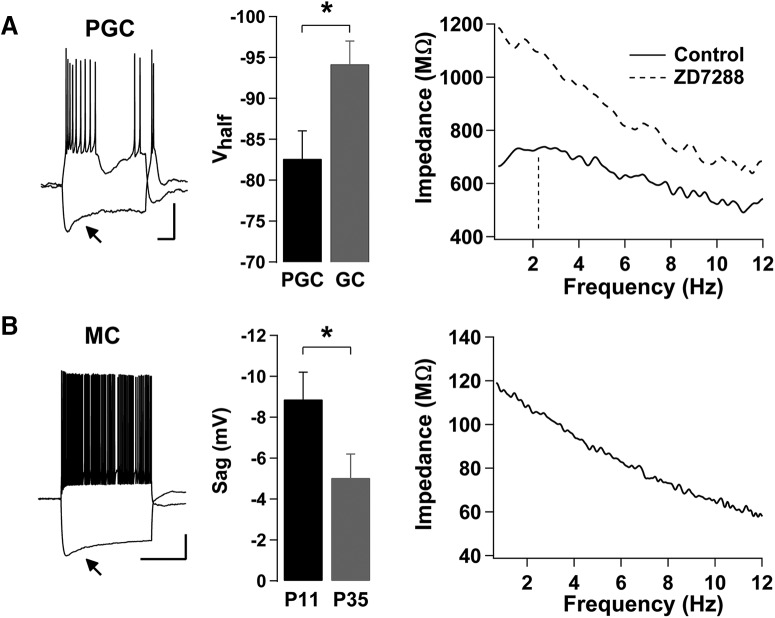
PGCs, but not MCs, exhibit *I*_h_-mediated subthreshold resonance in the MOB. ***A***, Left, Current-clamp responses of an MOB PGC; a hyperpolarizing current step (−10 pA) produces a sag in *V*_m_ (arrow), while a depolarizing current step (5 pA) elicits a burst of action potentials that is characteristic of PGCs. The resting *V*_m_ in this cell is −70 mV. Calibration: 20 mV and 200 ms. Middle, Summary bar graph showing that PGCs (*n* = 9) exhibit a more depolarized *V*_half_ than GCs (*n* = 11). Right, Impedance profile of a different PGC obtained using a ZAP protocol while holding at −70 mV. The subthreshold resonance is abolished in the presence of ZD7288 (control: black line, *f*_res_ = 2.24 Hz, indicated by the vertical dashed line, and Q = 1.10; ZD7288, dashed line, *f*_res_ = *f*_low_, and *Q* = 1). ***B***, Left, A current-clamp response of an MC at P11; a hyperpolarizing current step (−350 pA) produces a sag in *V*_m_ (arrow), while a depolarizing current step (350 pA) elicits a train of action potentials. The resting *V*_m_ in this cell is −60 mV. Calibration: 20 mV and 1 s. Middle, Summary bar graph showing the decrease in sag potential in MCs between P11 and P35 (P11, *n* = 8; P35, *n* = 6). Right, Impedance profile of the MC shown on the left, obtained using a ZAP protocol while held at −90 mV. Despite exhibiting *I*_h_, the cell is nonresonant (*f*_res_ = *f*_low_, and *Q* = 1).

We next examined whether other neurons in the OB circuit exhibit subthreshold resonance. In particular, we examined another inhibitory neuron, the PGC, which contributes to network oscillations entrained by the respiratory cycle (Fukunaga et al., 2014). Therefore, we examined the presence of subthreshold resonance in PGCs sampled from animals older than P30. MOB PGCs exhibited a prominent *I*_h_, with a sag potential of 5.2 ± 1.5 mV (*n* = 9). In recordings conducted with cAMP in the pipette, the *V*_half_ for *I*_h_ in PGCs was more positive than in MOB GCs (Fig. 7*A*; −82.6 ± 3.4 mV, *n* = 9; Student’s *t* test, *p* < 0.03), while τ was significantly slower (81.1 ± 22.5; *n* = 9; Student’s *t* test, *p* < 0.01), indicating differences in *I*_h_ between these inhibitory subtypes. Importantly, the majority of MOB PGCs (9 of 11 PGC) exhibited subthreshold resonance with a mean resonant frequency of 2.02 ± 0.16 Hz and a *Q* factor of 1.15 ± 0.03. Moreover, application of ZD7288 completely abolished the subthreshold resonance (Fig. 7*A*; *n* = 3), indicating that subthreshold resonance in PGCs, like in GCs, depends on the expression of *I*_h_.

In addition, we examined subthreshold resonance in MOB MCs, which have been previously shown to have *I*_h_, with its expression decreasing with age (Angelo and Margrie, 2011; Yu et al., 2015). To this extent, we recorded from two age groups of MCs, young (P11; *n* = 8) and old (P35; *n* = 6). In both age groups, MCs exhibited *I*_h_, and, in agreement with previous work, *I*_h_ was more prominent in the younger group, with sag values of 8.9 ± 1.3 mV (young) and 5.0 ± 1.2 mV (old; Fig. 7*B*; Student’s *t* test, *p* < 0.02). In these recordings, which we conducted with cAMP in the pipette, the *V*_half_ for *I*_h_ was −97 ± 2.8 mV, and τ was 500 ± 84 ms at P11 (MC τ vs PGC τ, Student’s *t* test, *p* < 0.0001). Intriguingly, despite the expression of *I*_h_, none of the recorded MCs, in either group exhibited subthreshold resonance. Furthermore, while *I*_h_ has also been observed in AOB MCs (data not shown; but see Gorin et al., 2016), we did not observe subthreshold resonance in recorded AOB MCs (*n* = 5) nor in AOB PGCs (*n* = 4).

## Discussion

Throughout the brain, the regulation of membrane excitability by *I*_h_ plays an important role in neuronal function and network dynamics (Lüthi and McCormick, 1998; Narayanan and Johnston, 2007; George et al., 2009; Benarroch, 2013). Network synchrony dynamics are an important component of the initial processing of odor signals in the OB. GCs comprise the largest population of inhibitory neurons in the OB, where they integrate local and afferent top–down signals to convey inhibition to output neurons, the MCs. Here, we show that *I*_h_ is present in GCs and that its expression follows the postnatal development of GCs, increasing with age. In addition, we show that GCs and MOB PGCs exhibit an *I*_h_-dependent subthreshold resonance in the 1–12 Hz frequency range. These results suggest that the regulation of *I*_h_ can play a role on circuit dynamics in the OB entrained by respiration.

GCs are a heterogeneous group of neurons, and they exhibit distinct characteristics in the AOB and MOB, including synaptic properties and regulation by neuromodulatory transmitters (Castro et al., 2007; Zimnik et al., 2013; Burton and Urban, 2015; Smith et al., 2015). Accordingly, we found that the voltage sensitivity of *I*_h_ was modulated by cAMP in the MOB, but not in the AOB; nevertheless, *I*_h_ activation was faster in the AOB in the presence of cAMP. Among HCN subunits, HCN1 exhibit the fastest kinetics, but the weakest cAMP sensitivity (Kaupp and Seifert, 2001; Accili et al., 2002), while HCN2 and HCN4 exhibit slower kinetics but are more cAMP sensitive (Kaupp and Seifert, 2001; Wainger et al., 2001). On the other hand, the HCN3 subunit exhibits slow kinetics but is not cAMP sensitive (Mistrik et al., 2005). Therefore, the kinetic parameters that we determined suggest that MOB GCs may express a combination HCN1, HCN2, and HCN4 subunits, while HCN3 subunits predominantly contribute to *I*_h_ in AOB GCs. In agreement with this possibility, all four HCN subunits are expressed in the OB, with HCN1 present in PGCs and GCs (Holderith et al., 2003; Fried et al., 2010), whereas HCN2 to HCN4 are more strongly expressed in the inner layers of the MOB (Notomi and Shigemoto, 2004). In the AOB, HCN1, HCN2, and HCN4 are moderately expressed, while HCN3 exhibits the strongest expression (Notomi and Shigemoto, 2004). Interestingly, despite exhibiting different kinetics and voltage sensitivity, our data with ZD7288 suggest that *I*_h_ in GCs of both regions is active at rest. In other neurons, *I*_h_ has been shown to contribute to baseline membrane excitability as well as dendritic integration (Magee, 1998; Fan et al., 2005; Benarroch, 2013); we propose a similar function of *I*_h_ in GCs. In fact, as they lack axons, the integration of synaptic inputs and the output occurs solely in dendritic processes in GCs. Interestingly, GCs receive most afferent excitatory input in basal and proximal dendrites (Boyd et al., 2012; Markopoulos et al., 2012); therefore, the extent of regulation by these inputs of dendrodendritic synapses in distal regions of GC's apical dendrites could also be modulated by the tonic influence of *I*_h_.

Mature AOB and MOB GCs exhibited subthreshold resonance, but there was a stark contrast in the membrane potential at which the resonance was strongest, as well as the peak frequency. In AOB GCs, subthreshold resonance was stronger at more depolarized potentials, and GCs were resonant in the delta range (1–4 Hz), whereas in MOB GC resonance was strongest at more negative potentials and resonance frequencies were in the theta range (4–12 Hz). These differences in GCs could underlie different circuit dynamics to accommodate their specific sensory inputs, which could be arriving at these specific frequency bands. In the MOB, inputs from the main olfactory epithelium are driven by breathing and arrive in the theta range (Smear et al., 2011; Manabe and Mori, 2013). Thus, in the theta range, subthreshold resonance for GCs may act as a bandpass filter for inputs at this frequency. In the AOB, however, the VNO is driven by the constriction and dilation of large blood vessels acting as a pump (Keverne, 1999); therefore, activity in the AOB may not be directly driven by breathing, but instead by the autonomic nervous system. Interestingly, previous studies (Dibattista et al., 2008; Cichy et al., 2015) have shown the presence of *I*_h_ in VNO sensory neurons; however, at this time it is unknown whether these cells exhibit subthreshold resonance. The lower frequency in AOB GCs suggests that the information processing and timing of inputs can work on a different timescale compared with the MOB. For example, the delta range resonance that we found in the AOB matches the lower-frequency baseline activity of AOB MCs near ∼4 Hz (Luo et al., 2003), as well as VNO activity near ∼1 Hz (Meredith, 1994). We note that differences in resonant frequency could be due to the contribution of other currents to the subthreshold resonance, for example, the M-current (*I_m_*), another non-inactivating potassium current associated with the KCNQ family of ion channels (Hutcheon and Yarom, 2000; Hu et al., 2009); however, our data indicate that in the MOB the subthreshold resonance arises from *I*_h_ and passive properties of GCs. Further studies should examine the possibilities of other contributing currents known to play a role in subthreshold resonance, such as *I*_m_, or *I*_NaP_, and corroborated with a computational model.

In several brain regions, *I*_h_ underlies the expression of subthreshold resonance in principal neurons, as well as interneurons (Hutcheon and Yarom, 2000; Ulrich, 2002, 2014; Hu et al., 2009; Zemankovics et al., 2010; Vera et al., 2014). Our data indicate that inhibitory neurons in the MOB, the GC and PGCs, exhibit subthreshold resonance and that this property is dependent on the presence of *I*_h_ in both subtypes; however, in the AOB, *I*_h_-mediated subthresold resonance may be present only in GCs. In fact, we found that subthreshold resonance in GCs strongly correlated with the presence of *I*_h_. This was particularly evident when we compared *I*_h_ expression and the presence of resonance during postnatal development. In 2-week-old GCs, *I*_h_ was smaller and the resonance strength was low, while they were significantly larger at 6 weeks. Although we did not perform a developmental analysis in MOB PGCs, we hypothesize that they exhibit a similar developmental progression of *I*_h_ size and the expression of subthreshold resonance. However, this was not the case for principal neurons, the MCs. In agreement with a previous report (Yu et al., 2015), we observed a developmental decrease in *I*_h_, yet, even when *I*_h_ was present, MCs did not exhibit *I*_h_-mediated subthreshold resonance. However, it has been suggested that MOB MCs exhibit gamma-frequency subthreshold resonance due to a persistent Na^+^ current (Brea et al., 2009), which was not addressed in our studies. Even in the AOB, *I*_h_ has been described in MCs, but blocking it had no effect on the intrinsic oscillations of MCs (Gorin et al., 2016). In agreement with these findings, we found no *I*_h_-mediated subthreshold resonance in AOB MCs.

In summary, we provide evidence that the two predominant subtypes of inhibitory neurons in the MOB, the GCs and PGCs, exhibit subthreshold resonance mediated by *I*_h_, but the primary output neurons, the MCs, do not. Various studies indicate that top–down neuromodulatory projections and cortical feedback principally target PGCs and GCs. Therefore, it is possible that subthreshold resonance and underlying *I*_h_ contribute to the integration of this top–down information. In addition, the activation of cAMP-dependent pathways by neuromodulators could lead to changes in *I*_h_ kinetics, affecting the resonant frequency and oscillations at the network level. Future *in vivo* studies will address how these properties of inhibitory neurons may contribute to OB circuit dynamics and odor processing.

## Synthesis

The decision was a result of the Reviewing Editor Anna Menini and the peer reviewers coming together and discussing their recommendations until a consensus was reached. A fact-based synthesis statement explaining their decision and outlining what is needed to prepare a revision is listed below. The following reviewer(s) agreed to reveal their identity: Graeme Lowe

Reviewer 1

The manuscript entitled "Hyperpolarization-activated currents and subthreshold resonance in granule cells of the olfactory bulb" reports the interesting finding that, in the mouse olfactory bulb, hyperpolarization-activated currents (Ih) are present in granule cells (GCs), which represent the most abundant olfactory bulb neuron type. Moreover, the authors show data that support a causal function of Ih in GC subthreshold resonance. Intriguingly, Ih properties differ between GCs in the main and accessory olfactory bulb (MOB / AOB) and both populations of inhibitory neurons appear to depict subthreshold resonance in different frequency ranges. Finally, using an elegant electroporation approach to express GFP specifically in postnatally born neurons the authors, the authors provide some evidence that Ih size and the strength of GC subthreshold resonance undergoes some postnatal developmental progression.

The authors use patch-clamp recordings in olfactory bulb slices to analyze both Ih properties and subthreshold resonance in GCs. Cell identity is confirmed by fluorescent dye labeling. In both voltage- and current clamp recordings, Ih is isolated by its 'unconventional' voltage-dependence of activation, its unique kinetic properties, its ion selectivity / permeability, and its pharmacology.

The findings presented by the authors are of interest to a wide range of individuals working in the field of (chemo)sensory neuroscience. The data are generally solid and the authors provide evidence for many of their claims. However, a few concerns - most of them minor - should be addressed before publication in eNeuro (see below).

Concerns:

a) My major concern is the interpretation of the data presented in Figure 7. The authors investigate four distinct time points post electroporation, i.e., 1, 2, 4, and 6 weeks. It is clear that there seems to be a shift in Ih expression and properties that occurs between week 1 and 2 post gene transfer. However, it is far less obvious whether or not there are any changes between weeks 2 and 6. In fact, one week after electroporation in the SVZ, the authors might record from neurons that are still in an immature state and not yet integrated in the olfactory bulb network. Obviously, as can be seen from Figure 7, most neurons at week 1 do not express any detectable Ih. Here, the authors should show original traces to confirm that the minute currents recorded are in fact Ih. Moreover, I do not concur with the interpretation (Discussion, p.19;.. 374-376) that "in two weeks old GCs Ih, if present, was very small and the resonance strength was low, while they were significantly larger at older ager (4-6 weeks)." Do the authors refer to the 1-week age group? In this general context, it is of particular concern that "recordings of GCs at negative potentials in 1 week old GCs were unstable..." Given the above mentioned uncertainties, I would suggest to not dwell on ontogenetic differences (provided that there is no robust effect between weeks 2 and 6 as the data appear to suggest) and, accordingly, delete the findings presented in Figures 6 (see below) and 7. This would make the study a more focused and concise short report.

b) Materials &amp; Methods, p.6, ll. 116-120: "In whole-cell recordings the internal solution had the following composition (in mM): 120 K-gluconate, 10 Na-Gluconate, 4 NaCl, 10 HEPES-K, 10 Na phosphocreatine, 2 Na-ATP, 4 Mg-ATP, and 0.3 GTP adjusted to pH 7.3 with KOH."

This amounts to a unusually increased Na+ concentration in the standard pipette solution (26 mM). Please clarify your rationale for using such high Na+ levels.

c) Results, p.11; ll. 224-228: "Across the total population of GC studied (AOB and MOB) resonant cells had larger Ih than non resonant cells, suggesting a correlation between Ih amplitude and subthreshold resonance (Fig 4C; resonant cells, Imax = -97.5 {plus minus} 10.3 pA, n= 25; non-resonant cells, Imax = -61 {plus minus} 13.0 pA, n= 15, p greater than 0.02)."

This can and should be analyzed. The data is there. I suggest a correlation analysis of Imax and Q factor (as shown in Fig. 4C).

d) Results, pp. 12-13: The authors investigated examined subthreshold resonance in MOB mitral cells, but not in AOB principle neurons. Why? One strength of their study is that we learn about cellular differences between AOB and MOB cell populations.

e) The statistical tests that were used need to be specified for each figure.

Minor points:

1) Introduction, p.3, ll. 49-53: "The main olfactory bulb (MOB) receives input from the nasal epithelium and is largely tuned to environmental odors, whereas the accessory olfactory bulb (AOB) receives input from the Vomeronasal organ and is responsible for pheromonal detection (Keverne, 1999; P. Lledo, Gheusi, &amp; Vincent, 2005; Shepherd, 1972).

In light of many recent findings, this distinction is much less absolute. Please rephrase.

2) Introduction, p.3, ll. 53-55: "In both AOB and MOB network synchrony is a substrate to important aspects of olfactory coding such as sparsening and feature binding of odor representations (Binns &amp; Brennan, 2005; Brody, 2003; Osinski &amp; Kay, 2016)."

The role of network synchrony is much less clear for the AOB than for the MOB. Whether, indeed, stimuli representations in the AOB show elements such as sparsening and feature binding remains to be determined.

3) Materials &amp; Methods, p.6, l. 106: "...temperature, or at 32 plus minus 2degree signC, using..."

I guess this is just a typo and the authors mean 37degree signC. If this is not the case, why using this somewhat 'intermediate' temperature?

4) Results, p.9: Fig. 2A is never mentioned in the main text.

5) Results, Figures 1-4 and 6: Throughout the text and captions, the authors refer to the data that is depicted in orange color as "red", which is quite confusing.

6) Results, p.10: the text mentions Figure 3A; however, Figure 3 has no panels...

7) Results, p.12: "...young (P11; n = 8) and old (P35 weeks; n = 6)."

Do the authors mean P35 or 35 weeks?

8) Results, p.13; l. 255: "...recordings, which we conducted with cAMP in the pipette, the..."

Why do these recordings under non-control conditions? This makes it difficult to interpret the results, especially since the authors have reported themselves that cAMP can have different effects.

9) Results, p.14; ll. 290-291: "...as a function of cell age (Fig. 7C).

There is no panel 7C.

10) Discussion, p.17: Some of the authors findings from AOB neurons should be discussed in the context of recently published data from Gorin et al. (2016, J. Neurosci.). There are some interesting similarities / parallels with respect to slow frequencies, lack of subthreshold Ih in AOB mitral cells, etc.

11) Figure 1B: The 'tail currents' upon return to -60 mV are rather puzzling. There should be no such current for the hyperpolarization to -70 mV. I would also guess that inactivation should change as a function of pre-pulse potential, but this appears not to be the case. Please clarify.

12) Figure 1: For the experiments under conditions of Ih block by ZD7288, the authors should show some offline subtracted traces to isolate the ZD7288-sensitive current.

13) Figure 1: Only data from AOB neurons is shown. It would nice to see some MOB GC data for comparison (holds true for Figure 2 as well).

14) Figure 2A, bottom panel: I do not understand what exactly is shown here and how these 'tail currents' were measured. Materials &amp; Methods and the corresponding caption do not really help.

15) Figure 3: How exactly was ΔV determined? Please elaborate.

16) Figure 3; original recording (top): Please show a similarly long control recording. Is there no change in Vm under control conditions?

17) Manuscript text: there are a number of typos and some grammar issues that should be addressed in a revised manuscript.

Reviewer 2

This study uses slice electrophysiology and pharmacology to demonstrate and characterize Ih currents, mediated by hyperpolarization-activated cyclic nucleotide gated channels, in a subpopulation of granule cells of both main (MOB) and accessory (AOB) olfactory bulbs of mouse. Mitral and periglomerular cells of MOB were also found to have Ih currents, but only the latter also exhibited low frequency resonance. Ih was active at rest and generated low frequency resonance in membrane voltage which the authors suggest contributes to oscillatory dynamics of bulb circuits entrained by periodic stimulus inputs in matching frequency ranges. The prevalence, kinetics and voltage/ cAMP-dependence of Ih differed between MOB and AOB granule cells, indicating functional specializations of the two different sensory pathways. In MOB, Ih and resonance increased progressively with development and maturation in postnatal newborn cells originating from the subventricular zone.

These findings provide novel information about granule cell biophysics that is important for understanding cellular and network dynamics in the MOB and AOB during odor signal processing. They are expected to significantly impact the field of olfactory bulb neurophysiology, and future computational studies will need to include granule cell Ih and resonance properties into their modeling. The experiments are well done with clean results and the paper well written and edited.

There are a few points to address.

(p. 6, lines 116-120): on the Na+ content of the pipette solutions: at negative resting potential close to calculated Ek = -104 mV, the major charge carrier for Ih is Na+. The apparently high [Na+]i = 26 mM translates to ENa = 46.5 mV at 32degree signC, and a driving force of -126.5 mV; contrast this to a more 'normal' neuronal [Na+]i = 10 mM which yields ENa = 152 mV. Thus, the cited pipette Na+ would cause Ih to be reduced to 83% of physiological current. Moreover, it is unclear if 'Na phosphocreatine' is really monosodium or disodium salt. Common commercially available formulations are Na2-phosphocreatine (e.g. Sigma, Tocris). Disodium salt would mean Na+i = 36 mM, Ena = 38 mV, and Ih reduced to 77% of physiological current. This could lead to significantly underestimating Ih and the strength of Ih-generated resonance.

(p. 10, lines 201-202): "perfusion of ZD7288 invariably produced a hyperpolarization in GCs (Fig 3A)" - how can one exclude the possibility that part of the membrane potential shift arises from presynaptic network effects of ZD7288? Should not this measurement be made under synaptic block? Do the spontaneous postsynaptic potentials change in frequency or amplitude when ZD7288 is applied?

(p. 11, lines 215-216): " The percentage of resonant cells was larger in the AOB than in the MOB; ∼71% (15/21) vs. ∼52% (10/19), respectively" - is this statement statistically correct? With modest sample sizes, the observed percentages may not be significantly different by standard statistical criteria. Consider the probability p(AOB) that the number of resonant AOB cells = 15 in a sample of 21 cells, if it is assumed that 52% of cells are resonant? From the binomial distribution of 2 outcomes (resonant vs. non-resonant): p(AOB) = SUM (r = 15 ... 21) (21!/((21-r)!*r!))*0.52^r * 0.48^(21-r) = 0.0574, so the observed AOB fraction is not significantly different from 52% at the 0.05 level of confidence. Conversely, the probability p(MOB) that the number of resonant MOB cells = 10 in a sample of 19 cells, if it is assumed that 71% of cells are resonant, is given by: p(MOB) = SUM (r = 1 ... 10) (19!/((19-r)!*r!))*0.71^r * 0.29^(19-r) = 0.0694, so the observed MOB fraction is also not significantly different from 71% at the 0.05 level.

(p. 11, lines 221-222): " these action potentials occurred within the range of fres for each cell (Fig 4B)." - this result needs to be quantified by statistical analysis of the action potential data.

(p. 12, lines 239-240): " we examined the presence of subthreshold resonance in PGCs sampled from animals older than P30" - the Materials and Methods section needs to

describe how PGC were identified among the heterogeneous population of juxtaglomerular neurons, i.e. specify the morphological or electrophysiological criteria used for identifying PGC. And Fig. 5 should show an Alexa 594 filled PGC, like Fig. 1A does for GC.

(p. 13, lines 257-258): " Intriguingly, despite the expression of Ih, none of the recorded MCs, in either group, exhibited subthreshold resonance" - for MC the Ih time constant (T=500 ms) is 6.2-fold slower than for PGC (T = 81 ms). If the resonant frequency fres is determined by T, then we might predict a 6.2-fold lower MC fres compared to PGC, i.e. 2.02 Hz/6.2 = 0.32 Hz, which is very close to the lower limit (0.2 Hz) of the FM sweep (ZAP) frequency range. Could this be why a resonant peak does not show up clearly?

(p. 14, lines 281-282): " Ih amplitude and Ri varied inversely during maturation (Fig. 7A)." - Fig 7A (right panel) is a strange way of plotting the data. It would be much more transparent to plot Ih amplitude vs. conductance (1/Ri), and the conductance should then increase linearly with Ih, right?

(p. 14, lines 290-291): " resonance in GCs increased as a function of cell age (Fig. 7C)." - Figure 7C does not exist, should it be 7B?

(p. 14, lines 292-295): " recordings of GCs at negative potentials in 1 week old GCs were unstable, however even using more positive holding potentials and larger a ZAP Vm amplitude, we found no evidence for subthreshold resonance at this age" - the lack of any resonance, or only very weak resonance at early age of GCs greater than 2 weeks, seems to contradict the authors suggestion that " Ih in GCs may contribute to the entrainment of the OB circuit to the respiratory rate and oscillatory sensory inputs during early olfactory-mediated behaviors (p. 1, lines 19-21)".

Actually, the logic and design of this experiment is confusing, if it is supposed to be concerned with neural mechanisms of early olfactory-mediated behaviors. The authors label neuroblasts in the SVZ postnatally and track their development of Ih, as they initially arrive in the MOB at 1 week, and then mature and become integrated into the bulbar circuitry several weeks subsequently. So these postnatally labeled cells would be unlikely to contribute to very early (greater than 2 weeks) sensory processing in newborn pups that "are strongly dependent on olfactory signaling to establish early maternal bonding".

(p. 16, lines 310-31): "These results suggest that regulation of Ih can play a role on circuit dynamics in the OB entrained by the respiration" - this may be true for PGC, but the suggestion that GC low frequency resonance plays a role in slow theta frequency oscillatory circuit dynamics, entrained by respiration, seems to be at odds with the findings of Fukunaga et al 2014, who showed that after optogenetic silencing of >85% of GC activity, "slow rhythms in MTC were unaffected and preferred phases were unaltered" (c.f. Fukunaga et al 2014: 3).

## References

[B1] Accili EA, Proenza C, Baruscotti M, DiFrancesco D (2002) From funny current to HCN channels: 20 years of excitation. News Physiol Sci 17:32–37. 11821534 10.1152/physiologyonline.2002.17.1.32

[B2] Angelo K, Margrie TW (2011) Population diversity and function of hyperpolarization-activated current in olfactory bulb mitral cells. Sci Rep 1:50. 10.1038/srep00050 22355569 PMC3216537

[B3] Benarroch EE (2013) HCN channels: function and clinical implications. Neurology 80:304–310. 10.1212/WNL.0b013e31827dec42 23319474

[B4] Binns KE, Brennan PA (2005) Changes in electrophysiological activity in the accessory olfactory bulb and medial amygdala associated with mate recognition in mice. Eur J Neurosci 21:2529–2537. 10.1111/j.1460-9568.2005.04090.x 15932610

[B5] Boyd AM, Sturgill JF, Poo C, Isaacson JS (2012) Cortical feedback control of olfactory bulb circuits. Neuron 76:1161–1174. 10.1016/j.neuron.2012.10.020 23259951 PMC3725136

[B6] Brody CD, Hopfield JJ (2003) Simple networks for spike-timing-based computation: with application to olfactory processing. Neuron 37:843–852. 12628174 10.1016/s0896-6273(03)00120-x

[B65] Brea JN, Kay LM, Kopell NJ (2009) Biophysical model for gamma rhythms in the olfactory bulb via subthreshold oscillations. Proceedings of the National Academy of Sciences, 106(51), 21954–21959. 10.1073/pnas.0910964106 PMC2799880 19996171

[B7] Burton SD, Urban NN (2015) Rapid feedforward inhibition and asynchronous excitation regulate granule cell activity in the mammalian main olfactory bulb. J Neurosci 35:14103–14122. 10.1523/JNEUROSCI.0746-15.2015 26490853 PMC4683680

[B63] Cang J, Isaacson JS (2003) In vivo whole-cell recording of odor-evoked synaptic transmission in the rat olfactory bulb. The Journal of Neuroscience, 23(10), 4108–4116. 12764098 10.1523/JNEUROSCI.23-10-04108.2003 PMC6741073

[B8] Carleton A, Petreanu LT, Lansford R, Alvarez-Buylla A, Lledo PM (2003) Becoming a new neuron in the adult olfactory bulb. Nat Neurosci 6:507–518. 10.1038/nn1048 12704391

[B9] Castro JB, Hovis KR, Urban NN (2007) Recurrent dendrodendritic inhibition of accessory olfactory bulb mitral cells requires activation of group I metabotropic glutamate receptors. J Neurosci 27:5664–5671. 10.1523/JNEUROSCI.0613-07.2007 17522311 PMC6672756

[B10] Cichy A, Ackels T, Tsitoura C, Kahan A, Gronloh N, Söchtig M, Engelhardt CH, Ben-Shaul Y, Müller F, Spehr J, Spehr M (2015) Extracellular pH regulates excitability of vomeronasal sensory neurons. J Neurosci 35:4025–4039. 10.1523/JNEUROSCI.2593-14.2015 25740530 PMC6605571

[B11] Craven KB, Zagotta WN (2006) CNG and HCN channels: two peas, one pod. Annu Rev Physiol 68:375–401. 10.1146/annurev.physiol.68.040104.134728 16460277

[B12] Dibattista M, Mazzatenta A, Grassi F, Tirindelli R, Menini A (2008) Hyperpolarization-activated cyclic nucleotide-gated channels in mouse vomeronasal sensory neurons. J Neurophysiol 100:576–586. 10.1152/jn.90263.2008 18509074

[B64] Egger V, Svoboda K, Mainen ZF (2005) Dendrodendritic synaptic signals in olfactory bulb granule cells: local spine boost and global low-threshold spike. The Journal of Neuroscience, 25(14), 3521–3530. 15814782 10.1523/JNEUROSCI.4746-04.2005 PMC6725376

[B13] Fan Y, Fricker D, Brager DH, Chen X, Lu HC, Chitwood R. a, Johnston D (2005) Activity-dependent decrease of excitability in rat hippocampal neurons through increases in I(h). Nature Neuroscience 8:1542–1551. 10.1038/nn1568 16234810

[B14] Fried HU, Kaupp UB, Müller F (2010) Hyperpolarization-activated and cyclic nucleotide-gated channels are differentially expressed in juxtaglomerular cells in the olfactory bulb of mice. Cell Tissue Res 339:463–479. 10.1007/s00441-009-0904-9 20140458 PMC2838509

[B15] Fukunaga I, Herb JT, Kollo M, Boyden ES, Schaefer AT (2014) Independent control of gamma and theta activity by distinct interneuron networks in the olfactory bulb. Nat Neurosci 17:1208–1216. 24997762 10.1038/nn.3760 PMC4146518

[B16] George MS, Abbott LF, Siegelbaum SA (2009) HCN hyperpolarization-activated cation channels inhibit EPSPs by interactions with M-type K(+) channels. Nat Neurosci 12:577–584. 10.1038/nn.2307 19363490 PMC2674138

[B17] Gorin M, Tsitoura C, Kahan A, Watznauer K, Drose DR, Arts M, Mathar R, O'Connor S, Hanganu-Opatz IL, Ben-Shaul Y, Spehr M (2016) Interdependent conductances drive infraslow intrinsic rhythmogenesis in a subset of accessory olfactory bulb projection neurons. J Neurosci 36:3127–3144. 10.1523/JNEUROSCI.2520-15.2016 26985025 PMC6705527

[B18] Gschwend O, Abraham NM, Lagier S, Begnaud F, Rodriguez I, Carleton A (2015) Neuronal pattern separation in the olfactory bulb improves odor discrimination learning. Nat Neurosci 18:1474–1482. 26301325 10.1038/nn.4089 PMC4845880

[B19] Gutfreund Y, Yarom Y, Segev I (1995) Subthreshold oscillations and resonant frequency in guinea-pig cortical neurons: physiology and modelling. J Physiol 483:621–640. 10.1113/jphysiol.1995.sp020611 7776248 PMC1157807

[B20] Holderith NB, Shigemoto R, Nusser Z (2003) Cell type-dependent expression of HCN1 in the main olfactory bulb. Eur J Neurosci 18:344–354. 10.1046/j.1460-9568.2003.02756.x 12887416

[B21] Hu H, Vervaeke K, Storm JF (2002) Two forms of electrical resonance at theta frequencies, generated by M-current, h-current and persistent Na+ current in rat hippocampal pyramidal cells. J Physiol 545:783–805. 10.1113/jphysiol.2002.029249 12482886 PMC2290731

[B22] Hu H, Vervaeke K, Graham LJ, Storm JF (2009) Complementary theta resonance filtering by two spatially segregated mechanisms in CA1 hippocampal pyramidal neurons. J Neurosci 29:14472–14483. 10.1523/JNEUROSCI.0187-09.2009 19923281 PMC6665813

[B23] Hutcheon B, Yarom Y (2000) Resonance, oscillation and the intrinsic frequency preferences of neurons. Trends Neurosci 23:216–222. 10782127 10.1016/s0166-2236(00)01547-2

[B24] Kaupp UB, Seifert R (2001) Molecular diversity of pacemaker ion channels. Annu Rev Physiol 63:235–257. 10.1146/annurev.physiol.63.1.235 11181956

[B25] Kay LM (2014) Circuit oscillations in odor perception and memory Prog Brain Res 208:223–251. 24767485 10.1016/B978-0-444-63350-7.00009-7

[B26] Keverne EB (1999) The vomeronasal organ. Science 286:716–720. 10531049 10.1126/science.286.5440.716

[B27] Lagier S, Carleton A, Lledo PM (2004) Interplay between local GABAergic interneurons and relay neurons generates gamma oscillations in the rat olfactory bulb. J Neurosci 24:4382–4392. 15128852 10.1523/JNEUROSCI.5570-03.2004 PMC6729436

[B28] Larriva-Sahd J (2008) The accessory olfactory bulb in the adult rat: a cytological study of its cell types, neuropil, neuronal modules, and interactions with the main olfactory system. J Comp Neurol 510:309–350. 10.1002/cne.21790 18634021

[B29] Lepousez G, Lledo PM (2013) Odor discrimination requires proper olfactory fast oscillations in awake mice. Neuron 80:1010–1024. 10.1016/j.neuron.2013.07.025 24139818

[B30] Lepousez G, Valley MT, Lledo PM (2013) The impact of adult neurogenesis on olfactory bulb circuits and computations. Annu Rev Physiol 75:339–363. 10.1146/annurev-physiol-030212-183731 23190074

[B31] Leszkowicz E, Khan S, Ng S, Ved N, Swallow DL, Brennan PA (2012) Noradrenaline-induced enhancement of oscillatory local field potentials in the mouse accessory olfactory bulb does not depend on disinhibition of mitral cells. Eur J Neurosci 35:1433–1445. 10.1111/j.1460-9568.2012.08070.x 22487171

[B32] Lledo PM, Gheusi G, Vincent J (2005) Information processing in the mammalian olfactory system. Physiol Rev 85:281–317. 10.1152/physrev.00008.2004 15618482

[B33] Lledo PM, Alonso M, Grubb MS (2006) Adult neurogenesis and functional plasticity in neuronal circuits. Nat Rev Neurosci 7:179–193. 10.1038/nrn1867 16495940

[B34] Ludwig A, Zong X, Jeglitsch M, Hofmann F, Biel M (1998) A family of hyperpolarization-activated mammalian cation channels. Nature 393:587–591. 10.1038/31255 9634236

[B35] Lüthi A, McCormick DA (1998) H-current: properties of a neuronal and network pacemaker. Neuron 21:9–12. 9697847 10.1016/s0896-6273(00)80509-7

[B36] Luo M, Fee MS, Katz LC (2003) Encoding pheromonal signals in the accessory olfactory bulb of behaving mice. Science 299:1196–1201. 10.1126/science.1082133 12595684

[B37] Magee JC (1998) Dendritic hyperpolarization-activated currents modify the integrative properties of hippocampal CA1 pyramidal neurons. J Neurosci 18:7613–7624. 9742133 10.1523/JNEUROSCI.18-19-07613.1998 PMC6793032

[B38] Manabe H, Mori K (2013) Sniff rhythm-paced fast and slow gamma-oscillations in the olfactory bulb: relation to tufted and mitral cells and behavioral states. J Neurophysiol 110:1593–1599. 10.1152/jn.00379.2013 23864376

[B39] Markopoulos F, Rokni D, Gire DDH, Murthy VNV (2012) Functional properties of cortical feedback projections to the olfactory bulb. Neuron 76:16. 10.1016/j.neuron.2012.10.028 PMC3530161 23259952

[B40] McCormick DA, Pape HC (1990) Properties of a hyperpolarization-activated cation current and its role in rhythmic oscillation in thalamic relaty neurones. J Physiol 431:319–342. 10.1113/jphysiol.1990.sp018331 1712843 PMC1181775

[B41] Meredith M (1994) Chronic recording of vomeronasal pump activation in awake behaving hamsters. Physiol Behav 56:345–354. 7938248 10.1016/0031-9384(94)90205-4

[B42] Mistrik P, Mader R, Michalakis S, Weidinger M, Pfeifer A, Biel M (2005) The murine HCN3 gene encodes a hyperpolarization-activated cation channel with slow kinetics and unique response to cyclic nucleotides. J Biol Chem 280:27056–27061. 10.1074/jbc.M502696200 15923185

[B43] Narayanan R, Johnston D (2007) Long-term potentiation in rat hippocampal neurons is accompanied by spatially widespread changes in intrinsic oscillatory dynamics and excitability. Neuron 56:1061–1075. 10.1016/j.neuron.2007.10.033 18093527 PMC2430016

[B44] Notomi T, Shigemoto R (2004) Immunohistochemical localization of Ih channel subunits, HCN1-4, in the rat brain. J Comp Neurol 471:241–276. 10.1002/cne.11039 14991560

[B45] Osinski BL, Kay LM (2016) Granule cell excitability regulates gamma and beta oscillations in a model of the olfactory bulb dendrodendritic microcircuit. J Neurophysiol 116:522–539. 27121582 10.1152/jn.00988.2015 PMC4978795

[B46] Pape HC, McCormick DA (1989) Noradrenaline and serotonin selectively modulate thalamic burst firing by enhancing a hyperpolarization-activated cation current. Nature 340:715–718. 10.1038/340715a0 2475782

[B47] Petreanu L, Alvarez-Buylla A (2002) Maturation and death of adult-born olfactory bulb granule neurons: role of olfaction. J Neurosci 22:6106–6113. 12122071 10.1523/JNEUROSCI.22-14-06106.2002 PMC6757952

[B48] Price JL, Powell TP (1970) The morphology of the granule cells of the olfactory bulb. J Cell Sci 7:91–123. 5476864 10.1242/jcs.7.1.91

[B49] Shepherd GM (1972) Synaptic organization of the mammalian olfactory bulb. Physiol Rev 52:864–917. 4343762 10.1152/physrev.1972.52.4.864

[B50] Smear M, Shusterman R, O’Connor R, Bozza T, Rinberg D (2011) Perception of sniff phase in mouse olfaction. Nature 479:397–400. 10.1038/nature10521 21993623

[B51] Smith RS, Hu R, DeSouza a, Eberly CL, Krahe K, Chan W, Araneda RC (2015) Differential muscarinic modulation in the olfactory bulb. J Neurosci 35:10773–10785. 10.1523/JNEUROSCI.0099-15.2015 26224860 PMC4518052

[B52] Ulrich D (2002) Dendritic resonance in rat neocortical pyramidal cells. J Neurophysiol 87:2753–2759. 12037177 10.1152/jn.2002.87.6.2753

[B53] Ulrich D (2014) Subthreshold delta-frequency resonance in thalamic reticular neurons. Eur J Neurosci 40:2600–2607. 24891125 10.1111/ejn.12630

[B54] Valsecchi F, Ramos-Espiritu LS, Buck J, Levin LR, Manfredi G (2013) cAMP and mitochondria. Physiology (Bethesda) 28:199–209. 10.1152/physiol.00004.2013 23636265 PMC3870303

[B55] van Brederode JFM, Berger a. J (2011) GAD67-GFP+ neurons in the Nucleus of Roller. II. Subthreshold and firing resonance properties. J Neurophysiol 105:249–278. 10.1152/jn.00492.2010 21047931 PMC3023385

[B56] Vera J, Pezzoli M, Pereira U, Bacigalupo J, Sanhueza M (2014) Electrical resonance in the θ frequency range in olfactory amygdala neurons. PLoS One 9:e85826. 10.1371/journal.pone.0085826 24465729 PMC3897534

[B57] Wainger BJ, DeGennaro M, Santoro B, Siegelbaum S. a, Tibbs GR (2001) Molecular mechanism of cAMP modulation of HCN pacemaker channels. Nature 411:805–810. 10.1038/35081088 11459060

[B58] Winner B, Cooper-Kuhn CM, Aigner R, Winkler J, Kuhn HG (2002) Long-term survival and cell death of newly generated neurons in the adult rat olfactory bulb. Eur J Neurosci 16:1681–1689. 12431220 10.1046/j.1460-9568.2002.02238.x

[B59] Yamaguchi M, Mori K (2005) Critical period for sensory experience-dependent survival of newly generated granule cells in the adult mouse olfactory bulb. Proc Natl Acad Sci U S A 102:9697–9702. 10.1073/pnas.0406082102 15976032 PMC1157102

[B60] Yu Y, Burton SD, Tripathy SJ, Urban NN (2015) Postnatal development attunes olfactory bulb mitral cells to high frequency signaling. J Neurophysiol 114:2830–2842. 26354312 10.1152/jn.00315.2015 PMC4737413

[B61] Zemankovics R, Káli S, Paulsen O, Freund TF, Hájos N (2010) Differences in subthreshold resonance of hippocampal pyramidal cells and interneurons: the role of h-current and passive membrane characteristics. J Physiol 588:2109–2132. 10.1113/jphysiol.2009.185975 20421280 PMC2905616

[B62] Zimnik NC, Treadway T, Smith RS, Araneda RC (2013) α(1A)-Adrenergic regulation of inhibition in the olfactory bulb. J Physiol 591:1631–1643. 10.1113/jphysiol.2012.248591 23266935 PMC3624843

