# Erratum: Hu et al., “Hyperpolarization-Activated Currents and Subthreshold Resonance in Granule Cells of the Olfactory Bulb”

**DOI:** 10.1523/ENEURO.0457-23.2023

**Published:** 2023-12-14

In the article “Hyperpolarization-Activated Currents and Subthreshold Resonance in Granule Cells of the Olfactory Bulb,” by Ruilong Hu, Katie A. Ferguson, Christina B. Whiteus, Dimphna H. Meijer, and Ricardo C. Araneda, which published online October 27, 2016, some funding information was omitted from the article. The Neurobiology Course at the Marine Biological Laboratory was supported by National Institutes of Health Grant R25-NS-063307. The full acknowledgment statement appears below.

This research was supported by National Institutes of Health (NIH)-National Institute on Deafness and Other Communication Disorders Grant DCR01-DC009817 and NIH-National Institute on Aging Grant AG-049937A (to R.C.A); National Science Foundation-Graduate Research Fellowships Program/Division of Graduate Education Grant 1322106 (to R.H); and NIH-National Institute of Neurologic Disorders and Stroke Grant R25-NS-063307 (to K.A.F, C.B.W, and D.H.M)

